# Exosomal Thrombospondin-1 Disrupts the Integrity of Endothelial Intercellular Junctions to Facilitate Breast Cancer Cell Metastasis

**DOI:** 10.3390/cancers11121946

**Published:** 2019-12-05

**Authors:** Junyu Cen, Lingyun Feng, Huichuan Ke, Lifeng Bao, Lin Z. Li, Yoshimasa Tanaka, Jun Weng, Li Su

**Affiliations:** 1 Key Laboratory of Molecular Biophysics of Ministry of Education, College of Life Science and Technology, Huazhong University of Science and Technology, Wuhan 430074, China; cenjy@hust.edu.cn (J.C.); lingyun_feng@hust.edu.cn (L.F.); m201771756@hust.edu.cn (H.K.); D201177384@hust.edu.cn (L.B.); 2 Department of Radiology and Abramson Cancer Center, Perelman School of Medicine, University of Pennsylvania, Philadelphia, PA 19104, USA; linli@pennmedicine.upenn.edu; 3 Center for Medical Innovation, Nagasaki University, 1-7-1, Sakamoto, Nagasaki 852-8588, Japan; ystanaka@nagasaki-u.ac.jp; 4 Research Institute of Huazhong University of Science and Technology in Shenzhen, Shenzhen 518063, China

**Keywords:** breast cancer, exosome, endothelial intercellular junctions, transendothelial migration, thrombospondin-1 (TSP1)

## Abstract

Transendothelial migration of malignant cells plays an essential role in tumor progression and metastasis. The present study revealed that treating human umbilical vein endothelial cells (HUVECs) with exosomes derived from metastatic breast cancer cells increased the number of cancer cells migrating through the endothelial cell layer and impaired the tube formation of HUVECs. Furthermore, the expression of intercellular junction proteins, including vascular endothelial cadherin (VE-cadherin) and zona occluden-1 (ZO-1), was reduced significantly in HUVECs treated with carcinoma-derived exosomes. Proteomic analyses revealed that thrombospondin-1 (TSP1) was highly expressed in breast cancer cell MDA-MB-231-derived exosomes. Treating HUVECs with TSP1-enriched exosomes similarly promoted the transendothelial migration of malignant cells and decreased the expression of intercellular junction proteins. TSP1-down regulation abolished the effects of exosomes on HUVECs. The migration of breast cancer cells was markedly increased in a zebrafish in vivo model injected with TSP1-overexpressing breast cancer cells. Taken together, these results suggest that carcinoma-derived exosomal TSP1 facilitated the transendothelial migration of breast cancer cells via disrupting the intercellular integrity of endothelial cells.

## 1. Introduction

Metastatic breast cancer remains the leading cause of mortality [1]. The process of tumor cell invasion and migration is comprised of a series of complex cellular biological events: local invasion, intravasation, survival in the circulation, arrest at a distant organ site, extravasation, micrometastasis formation, and metastatic colonization, in which the penetration of vessel wall during intravasation of tumor cells is a critical step [2]. Intravasation can be facilitated by molecular changes that promote the ability of carcinoma cells to cross the endothelial cell barriers. For example, the cytokine-transforming growth factor-β (TGF-β) signaling can enhance intravasation, at least in part, through induction of epithelial-mesenchymal transition (EMT) [3]. Single and double small interfering RNA (siRNA)-mediated knockdown of S100 calcium-binding protein P (S100P) and Ezrin in the triple-negative breast cancer-derived cells significantly inhibited their transendothelial migration of malignant and destabilized the intercellular junctions of endothelial cells [4]. These processes are closely related to tumor microenvironments.

Tumor microenvironment comprises cells, such as vascular endothelial cells, fibroblasts, and lymphocytes, as well as extracellular vesicles and molecules, which are involved in tumor metastasis [5,6]. Whereas intravasation depends on the migration ability of tumor cells themselves, disruption of vascular barrier integrity can also promote tumor cell migration. Matrix metalloproteinase-1 (MMP-1), matrix metalloproteinase-2 (MMP-2), cyclooxygenase-2 (COX-2), and epiregulin (EREG) synergistically promote breast carcinoma intravasation by stimulating neoangiogenesis and leaky blood vessel formation [7]. Besides these molecules, extracellular vesicles in the tumor microenvironment play an essential role in transendothelial migration.

Cells can release extracellular vesicles of different intracellular origins, with the size ranging from a few dozens of nanometers to a few micrometers [8]. Exosome is one of the extracellular vesicles, classically defined by the small size (30–150 nm in diameter), secreted by various kinds of cells in the tumor microenvironment [9,10,11]. It has been demonstrated that tumor-derived exosomal proteins and RNA are involved in vascular angiogenesis and permeability [12,13], and also closely associated with transendothelial migration of tumor cells. In addition, exosomes derived from hypoxic leukemia cells enhance the tube formation in endothelial cells [14]. Cancer cell-derived exosomal microRNA-25-3p (miR-25-3p) regulates the expression of vascular endothelial growth factor receptor 2 (VEGFR2), zona occluden-1 (ZO-1), occludin and claudin5 in endothelial cells to promote vascular permeability and angiogenesis, and subsequently enhances the migration of colorectal cancer cells [15].

In the present study, we firstly examined the effect of breast cancer-derived exosomes on cancer cell intravasation and on the expression of junction molecules in human umbilical vein endothelial cells (HUVECs). We then set out to identify proteinaceous factors in the tumor-derived exosomes that were responsible for the alteration in the potential for tumor transendothelial migration. Proteomic analysis revealed that a high level of thrombospondin-1 (TSP1) was expressed in exosomes. We further demonstrated that the exosomal TSP1 facilitated the migration of breast cancer cells in vitro and in vivo. Overall, our work demonstrated a novel mechanism underlying the interaction between breast cancer cell-derived exosomes and endothelial cells that facilitates the transendothelial migration of breast cancer cells.

## 2. Results

### 2.1. Exosome-Containing Fraction Derived from Cancer Cells Enhances the Transendothelial Migration of Breast Cancer Cells

**Figure 1:**
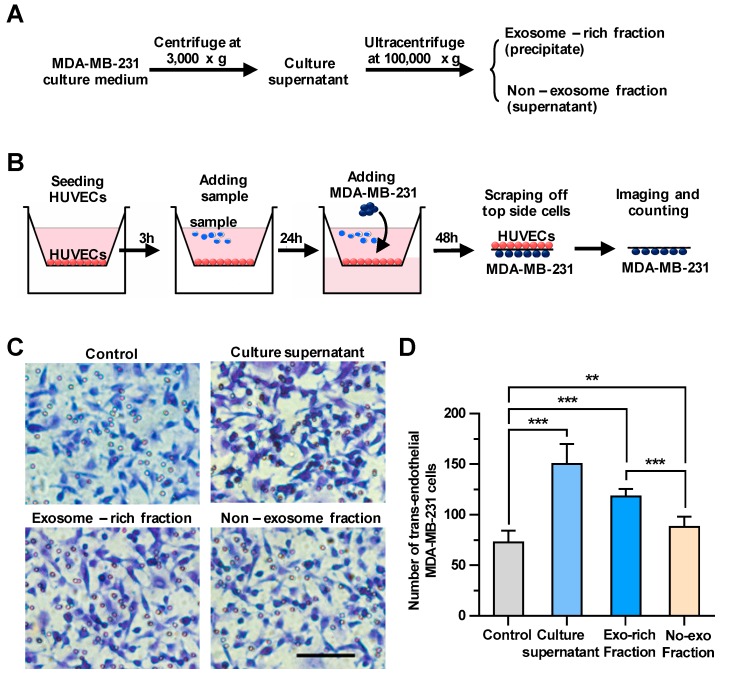
The cancer cell-derived culture supernatant and the exosome-enriched fraction significantly enhance the transendothelial migration of breast cancer cells. (**A**) A scheme for the preparation of the cell culture supernatant, the exosome-enriched fraction and non-exosome fraction. (**B**) A schematic diagram of the transendothelial migration assay. (**C**) The transendothelial migration of breast cancer cell MDA-MB-231 after treating human umbilical vein endothelial cells (HUVECs) with the culture supernatant, exosome-enriched precipitate or non-exosome supernatant fractions. Scale bar, 100 μm. (**D**) Quantitative analyses of the migrated MDA-MB-231. Three independent experiments were performed, in which cell numbers in eight photo images were counted. ** *p* < 0.01, *** *p* < 0.001 by unpaired Student’s *t*-test.

To explore whether the culture supernatant of breast cancer cells could regulate the transendothelial migration of carcinomas, we first harvested culture supernatants of MDA-MB-231, a metastatic human breast cancer cell line, as shown in Figure 1A. We then examined the effect of the culture supernatants on the integrity of endothelial cells using a transendothelial migration assay (Figure 1B). Cells migrated down to the bottom side of the filters were stained and counted under a microscope. In wells only with HUVECs, but without MDA-MB-231 cells, no cells were observed on the bottom side of the transmembrane (Figure S1), indicating that cells migrated down to the bottom side of the transmembrane should be MDA-MB-231 cells, but not HUVECs. As shown in Figure 1C,D, treating HUVECs with the culture supernatants significantly facilitated the transendothelial migration of breast cancer cells. Then, the rest of the culture supernatant was fractionated by ultracentrifugation into the culture supernatant and the exosomes-enriched precipitate, which were utilized for the same transendothelial migration assay (Figure 1A). It was demonstrated that treating HUVECs with both fractions could enhance the transendothelial migration of MDA-MB-231 cells, whereas the effect of the precipitate fraction enriched with exosomes on the tumor migration (increasing 63% by comparing to control) was more pronounced than that of the supernatant fraction without exosomes (increasing 21% by comparing to control), suggesting that breast cancer-derived exosomes could significantly enhance the transendothelial migration of MDA-MB-231 cells.

### 2.2. Exosomes Enhance the Transendothelial Migration of MDA-MB-231 Cells and Inhibit the Tube Formation in HUVECs

**Figure 2:**
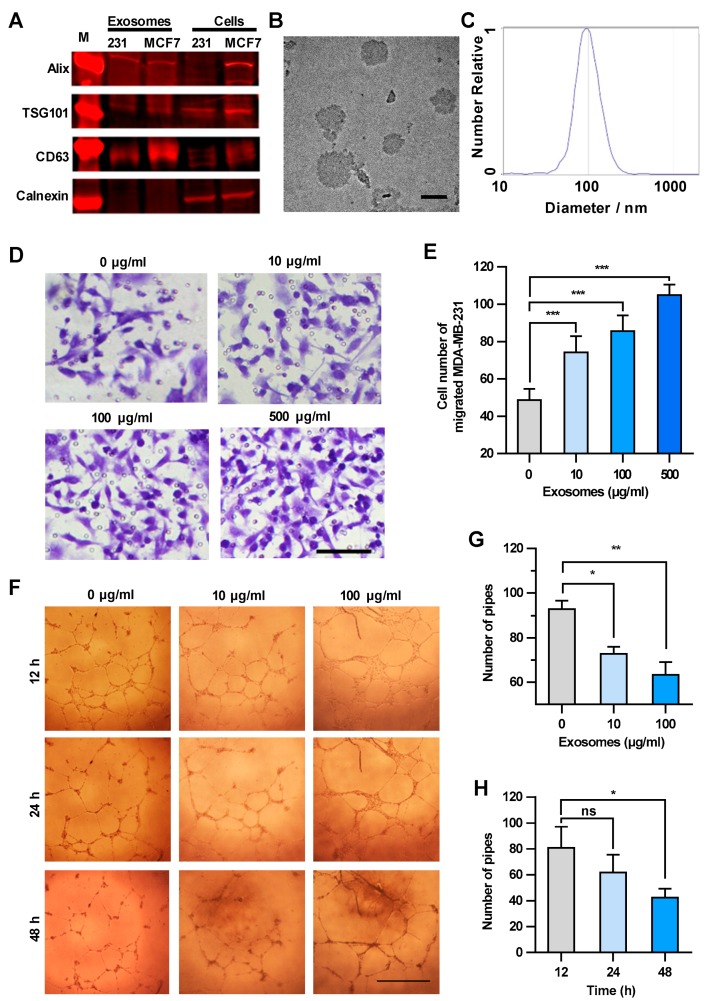
Exosomes enhance the transendothelial migration of breast cancer cells and inhibit the HUVEC tube formation. (**A**) Expression of exosome markers in MDA-MB-231 exosomes revealed by Western blot analysis. (**B**) An electron microscopic image of exosomes (red arrows) derived from MDA-MB-231. Scale bar, 100 nm. (**C**) Granularity and uniformity of exosomes determined by nanoparticle tracking analysis. (**D**) Images of transendothelial MDA-MB-231 cells after treating HUVECs with different concentrations of exosomes. Scale bar, 100 μm. (**E**) Migration of MDA-MB-231 through HUVECs layers after treatment with MDA-MB-231-derived exosomes. (**F**) Image of tube formation in exosome-treated HUVECs. Scale bar, 1 mm. (**G**) The tube formation in HUVECs treated with different concentrations of exosomes. (**H**) Time course of the tube formation in HUVECs treated with 100 μg/mL exosomes. Data are shown as means ± standard deviation (SD) and results were from three independent experiments. * *p* < 0.05, ** *p* < 0.01, *** *p* < 0.001; ns, no significance by unpaired Student’s *t*-test.

Based on the above results, cancer cell-derived exosomes are likely to be the main components that promote the transendothelial migration of MDA-MB-231 cells. Thus, the exosomes in the culture supernatant were isolated and characterized biologically and physically [16]. The expression of specific markers of exosomes, i.e., 3-mannosyltransferase (ALG-2)-interacting protein X (Alix), tumor susceptibility protein 101 (TSG101) and cluster of differentiation 63 (CD63), were confirmed in the exosome samples by Western blot analysis (Figure 2A). The sizes of the exosomes in the electron microscope image were approximately 100 nm in diameter (Figure 2B). The granularity and uniformity of the exosomes were determined by nanoparticle tracking analysis with a mean size of 94.4 nm in diameter. The size of 95% of the exosomes ranged from 45–165 nm and the concentration was 4.5 × 10^7^/mL (Figure 2C). These results indicated that the exosomes are the dominant components in the extracellular vesicle samples we prepared by using a Total Exosome Isolation Reagent kit.

We next examined the concentration-dependent effect of exosomes derived from MDA-MB-231 cells on the transendothelial migration of tumor cells. HUVECs were treated with increasing amounts of exosomes, and then MDA-MB-231 cells were seeded onto the HUVECs layer. The integrity of HUVEC intercellular junctions is the determinant factor of transendothelial migration of tumor cells [17,18]. As shown in Figure 2D,E, MDA-MB-231 cells migrating through the HUVECs layer increased in an exosome concentration-dependent manner, suggesting that the integrity of HUVEC intercellular junctions decreased after the treatment with cancer cell-derived exosomes. It is worth mentioning that the number of migrated MDA-MB-231 cells showed no significant changes after treatment with GW4869, which could inhibit exosome secretion from MDA-MB-231 cells, indicating that the exosomes added into the upper chamber showed dominant effect on the integrity of HUVECs (Figure S2). Since the endothelial tube formation defines the integrity of intercellular junctions between endothelial cells [19,20], we examined the effect of cancer cell-derived exosomes on the HUVEC tube formation. As shown in Figure 2F–H, MDA-MB-231-derived exosomes inhibited the HUVEC tube formation in both concentration-dependent and time-dependent manners. It is worthy of note that the exosomes did not alter the vitality of HUVECs less than the concentration of 500 μg/mL within 48 h (Figure S3). Taken together, exosomes secreted from MDA-MB-231 cells disrupted the integrity of the endothelial cell junctions and enhanced the transendothelial migration of breast cancer cells.

### 2.3. Exosomes Derived from MDA-MB-231 Cells Suppress the Expression of Intercellular Junction Proteins in HUVECs

**Figure 3:**
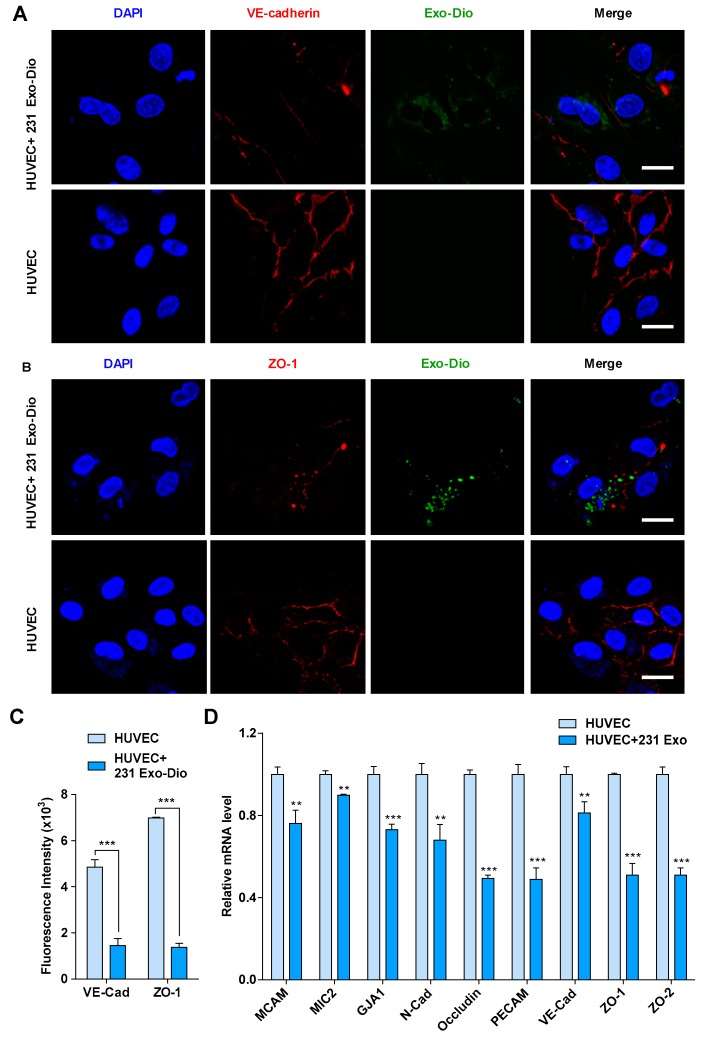
Exosomes derived from MDA-MB-231 cells regulate the expression of intercellular junction molecules in HUVECs. (**A**,**B**) Confocal microscopy images of vascular endothelial cadherin (VE-cadherin) (red) (**A**) and zona occluden-1 (ZO-1) (red) (**B**) in exosome-treated HUVECs stained with corresponding antibodies. Exosomes were labeled with Dio (green). Scale bar, 20 μm. (**C**) Mean fluorescence intensity for VE-cadherin and ZO-1 expression in exosome-treated and control HUVECs. (**D**) mRNA expression of intercellular junction molecules in exosome-treated and control HUVECs by qRT-PCR analysis. Data are shown as mean ± SD and representative of three independent experiments. ** *p* < 0.01, *** *p* < 0.001 by unpaired Student’s *t*-test.

It has been reported that the intercellular junction proteins play a pivotal role in the maintenance of the vascular endothelial integrity [21,22]. We thus examined the effect of MDA-MB-231-derived exosomes on the expression of vascular endothelial cadherin (VE-cadherin) and ZO-1, two representative components in intercellular junctions, by immunofluorescence imaging [23,24]. When HUVECs were treated with exosomes derived from MDA-MB-231, the expression of both VE-cadherin and ZO-1 was significantly suppressed (Figure 3A,B). Fluorescence intensity analyses confirmed the inhibition of their expression (Figure 3C), indicating that MDA-MB-231-derived exosomes disrupted the integrity of intercellular junction via suppression of junction protein expression. We next examined mRNA levels of some molecules involved in the formation of intercellular junctions, including melanoma cell adhesion molecule (MCAM), microneme protein-2 (MIC2), gap junction alpha-1 protein (GJA1), N-cadherin, occludin, platelet endothelial cell adhesion molecule-1 (PECAM), VE-cadherin, ZO-1, and zona occluden-2 (ZO-2), by quantificational real-time polymerase chain reaction (qRT-PCR). Upon treating HUVECs with MDA-MB-231-derived exosomes, the transcription of all the intercellular junction molecules was significantly reduced, of which occludin, PECAM, ZO-1, and ZO-2 were markedly reduced by 51%, 51%, 49%, and 49%, respectively (Figure 3D). Based on the above results, it is most likely that tumor-derived exosomes disrupt the integrity of intercellular junctions by suppressing the expression of junction molecules, leading to the loose interaction among endothelial cells.

### 2.4. Tumor-Derived Exosomal TSP1 Promotes the Transendothelial Migration of Breast Cancer Cells In Vitro

**Figure 4:**
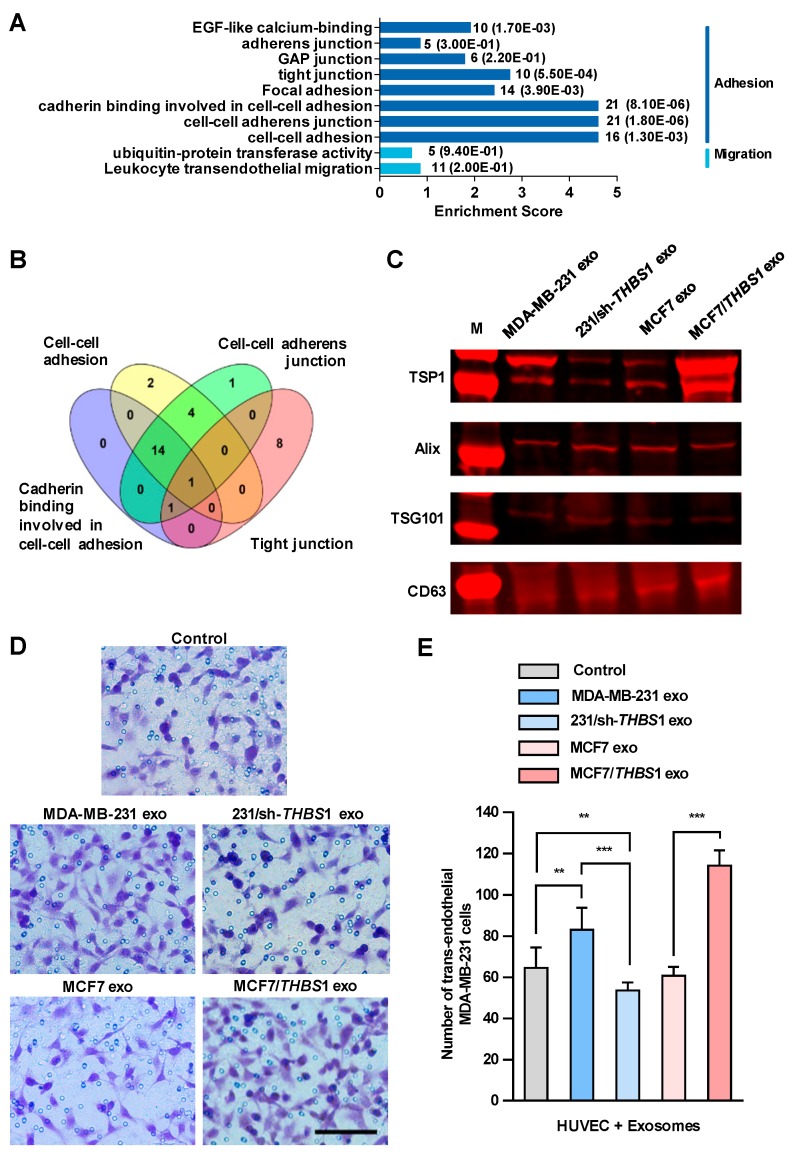
Exosomal thrombospondin-1 (TSP1) enhances the transendothelial migration of breast cancer cells. (**A**) The biological processes revealed by Gene Ontology (GO) analysis of exosomal proteome identified from mass spectral data. The numbers after each column indicated the hit number in each subcategory and *p*-value in parenthesis. (**B**) Overlapping of GO enriched biological processes for exosomal proteome related to cell–cell junction. The number of genes contained in each cluster is indicated. (**C**) TSP1 expression in exosomes derived from parent and gene-transfected breast cancer cells by Western blot analyses. (**D**) Transendothelial migration of MDA-MB-231 after treating HUVECs with exosomes derived from breast cancer cells differentially expressing TSP1. Scale bar, 100 μm. (**E**) Quantitative analyses of the migrated MDA-MB-231 cells in transwell assay. Data are shown as mean ± SD and representative of three independent experiments. ** *p* < 0.01, *** *p* < 0.001 by unpaired Student’s *t*-test.

We then set out to identify the factors in exosomes that could disrupt the intercellular integrity of HUVECs. Firstly, we conducted mass spectrometry analysis of the exosomes derived from MDA-MB-231 cells and a total of 1315 proteins were identified, which were classified into 315 protein groups (Table S1). We analyzed all protein candidates by Gene Ontology (GO) enrichment, and found that sixty protein candidates could participate in the biological processes related to adhesion and migration (Figure 4A). As depicted in Figure 4B, by overlying four GO enriched biological processes related to intercellular junction (“cadherin binding involved in cell–cell adhesion”, “cell–cell adhesion”, “tight junction” and “focal adhesion”), which had higher enrichment scores within the subcategories related to adhesion and migration, TSP1 was identified as a unique candidate appearing in all four processes. Additionally, TSP1 was the only protein candidate that matched 18 unique peptides identified by mass spectrometry, which should be the most relevant protein candidates in exosomes (Table S1).

We observed a high level of *THBS1* mRNA expressed in MDA-MB-231, the human gene encoding TSP1, whereas only a very low expression in MCF7. In addition, TSP1 was highly expressed in exosomes, but very low in MDA-MB-231 cells (Figure S4). Recent study showed that Ras-related protein Rab-37-mediated TSP1 secretion in cancer cells is associated with the inhibition of the migration and angiogenesis of surrounding endothelial cells in vitro and in vivo [25]. TSP1 is an important matricellular glycoprotein that mediates cell-to-cell and cell-to-matrix interactions in tumor microenvironment and is a potent inhibitor of angiogenesis [26]. TSP1 induces cell migration in several tumor cell lines, indicating that this protein may promote cancer invasion [27,28,29], whereas the physiological function of TSP1 in exosomes has not been fully elucidated yet. To clarify the function of TSP1 in exosomes released by breast cancer cells, we established MDA-MB-231/sh-*THBS1*, a TSP1-knockdown MDA-MB-231 cell line and MCF7/*THBS1*, a TSP1 stably expressing MCF7 cell line, and prepared exosomes derived from the transfectants as well as parent breast cancer cells (Figure 4C and Figure S5). As shown in Figure 4D,E, the transendothelial migration of MDA-MB-231 cells reduced significantly when HUVECs were treated with exosomes derived from TSP1-knockdown MDA-MB-231, compared to those from parent MDA-MB-231 cells. On the contrary, treating with exosomes derived from MCF7/*THBS1* markedly impaired the integrity of the endothelial layer and enhanced the transendothelial migration of MDA-MB-231 cells, when compared to MCF7-derived exosomes. In addition, we examined the transendothelial migration ability of MCF-7 and MCF-7/*THBS1* cells after treating with MCF-7 or MCF-7/*THBS1* exosomes and the data have been presented in Supplementary Figure S6. Our result validated that the migration ability of MCF7 cells increased when TSP1 was highly expressed, consisting with the previous publishes [28,30]. TSP1-enriched exosomes treatment could further enhance the transendothelial migration of MCF7 cells.

### 2.5. Exosomal TSP1 Derived from Breast Cancer Cells Is Responsible for the Suppression of Intercellular Junction Molecules

**Figure 5:**
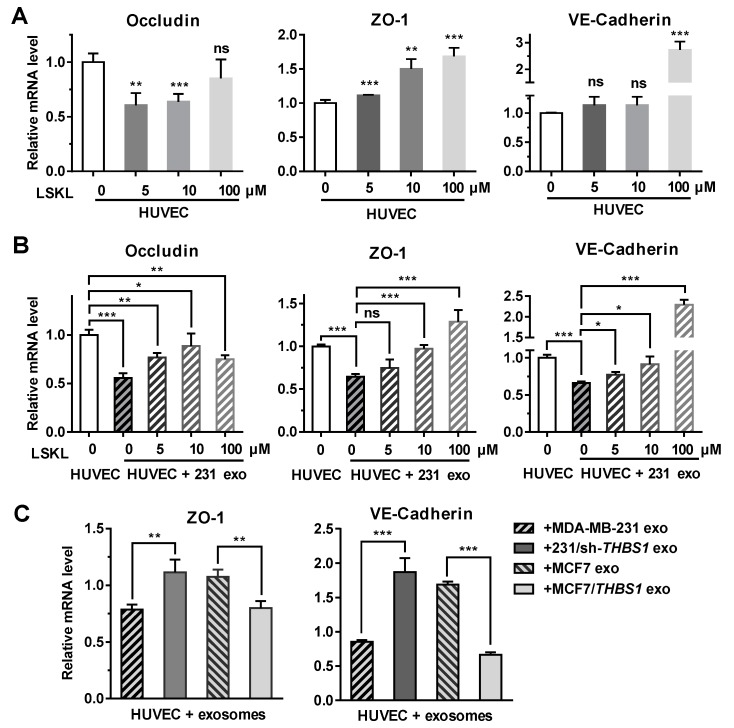
Exosomal TSP1 derived from breast cancer cells suppresses the expression of HUVEC intracellular junction proteins. (**A**) mRNA transcription level of occludin, ZO-1, VE-cadherin, in HUVECs treated with different amount of LSKL. (**B**) mRNA transcription level of occludin, ZO-1, VE-cadherin, in HUVECs treated with MDA-MB-231-derived exosomes and different concentrations of LSKL. (**C**) mRNA transcription level of ZO-1 and VE-cadherin in HUVECs treated with exosomes derived from breast cancer cells differentially expressing TSP1. Data are shown as mean ± SD and representative of three independent experiments. * *p* < 0.05, ** *p* < 0.01, *** *p* < 0.001; ns, no significance by unpaired Student’s *t*-test.

To further analyze if TSP1 could modulate the expression of intercellular junction molecules such as occludin, ZO-1 and VE-cadherin, the transcriptional level of these genes was examined in endothelial cells. As shown in Figure 5A, the expression of ZO-1 and VE-cadherin in HUVECs was enhanced in the presence of the TSP1-inhibitory peptide LSKL (leucine–serine–lysine–leucine) in a concentration-dependent manner. When HUVECs were treated with MDA-MB-231-derived exosomes, the transcription of occludin, ZO-1, and VE-cadherin was suppressed. Adding LSKL to the exosome-treated cells, however, restored the transcription of the ZO-1 and VE-cadherin in a LSKL concentration-dependent manner (Figure 5B), suggesting that TSP1 from exosomes should be responsible for the suppression of the ZO-1 and VE-cadherin mRNA transcription. By contrast, Figure 5A,B revealed that occludin transcription was likely not to be solely dependent on TSP1, because its transcription was not modulated by the LSKL peptide.

When HUVECs were treated with TSP1-knockdown MDA-MB-231-derived exosomes, the expression of ZO-1 and VE-cadherin were enhanced. In contrast, the ZO-1 and VE-cadherin mRNA transcription was upregulated after HUVECs were treated with MCF7-derived exosomes, whereas the exosomes derived from MCF7/*THBS1* cells significantly suppressed the mRNA expression (Figure 5C). The results further confirmed that TSP1 in the tumor cell-derived exosomes was responsible for the suppression of molecules maintaining the intercellular junctions of endothelial cells.

### 2.6. Exosomal TSP1 Promotes the Migration of Tumor Cells in Zebrafish

**Figure 6:**
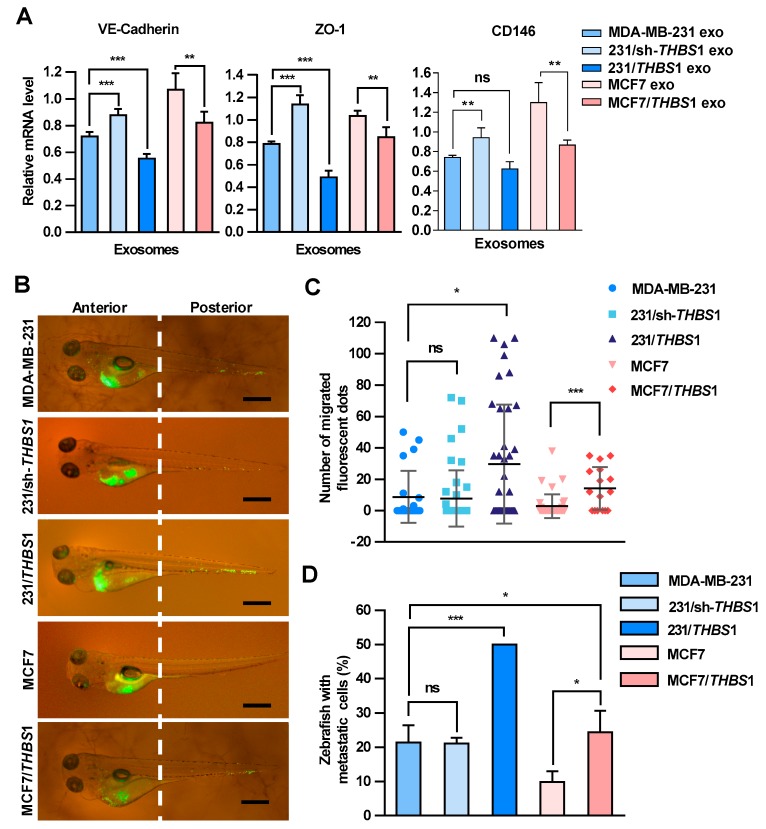
Carcinoma-derived exosomal TSP1 promotes the transendothelial migration of tumor cells in zebrafish. (**A**) Effect of exosomes derived from breast cancer cells on the expression of VE-cadherin, ZO-1 and CD146 mRNAs. Exosomes derived from MDA-MB-231 cells were injected into 48 h post fertilization (hpf) zebrafish embryos. At 48 h after the exosome injection, 30 embryos injected with exosomes and 30 control embryos were collected for mRNA level analysis by qRT-PCR. (**B**) Fluorescence imaging of the breast cancer cell migration in zebrafish. Dio-labeled breast cancer cells were injected into the yolk sac of zebrafish. Arrowheads indicate disseminated tumor foci (single tumor cells or cell aggregates) in the tail regions. Scale bar, 500 μm. (**C**) Quantitative analyses of the breast cancer cells located within the zebrafish tail region. (**D**) Proportion of zebrafish with the tumor cells migrated to the tail (%). Data are shown as mean ± SD and representative of three independent experiments. * *p* < 0.05, ** *p* < 0.01, *** *p* < 0.001; ns, no significance by unpaired Student’s *t*-test.

Zebrafish has been used as a useful vertebrate model to study metastatic processes of tumors [31]. To further examine the effect of TSP1 on the transendothelial migration of breast cancer cells, exosomes with various amounts of TSP1 were prepared from parent breast cancer cells and the transfectants (Figures S5 and S7), and then injected into zebrafish yolk sac. The expression of VE-cadherin, ZO-1, and CD146 in zebrafish was detected by qRT-PCR (Figure 6A). The results showed that exosomal TSP1 inhibited the mRNA expression level of VE-cadherin in zebrafish, which was specifically expressed in endothelial cells. Moreover, Dio-labeled breast cancer cells were injected into the zebrafish yolk sac and the migration of the tumor cells to the tail was monitored under a fluorescence microscope 48 h later (Figure 6B). It was clearly showed that the tail migration of tumor cells was more prominent for TSP1-overexpressing MDA-MB-231 cells compared to parent cells. Moreover, the tail metastasis proportion of the zebrafish group injected with tumor cells expressing a high level of TSP1 was significantly higher than that of other two groups (MDA-MB-231 and 231/sh-*THBS1*). Essentially the same phenomenon was found between MCF7/*THBS1* and MCF7 cell groups (Figure 6C,D). The results collectively demonstrated that breast cancer cells expressing a high level of TSP1 exhibited a high level of metastatic potential in vivo.

## 3. Discussion

Metastasis is a prominent feature during the progression of breast cancer and the main cause of death among breast cancer patients. Tumor microenvironment, comprising of various proteins, nucleic acids and vesicles, plays a key role in cancer progression to metastasis. TSP1 is one of the extracellular matrix glycoproteins, generally secreted by endothelial cells, fibroblasts, smooth muscle cells, and regulates the signaling pathways of CD47, CD36 and TGF-β [32,33]. The present study demonstrated that TSP1 is one of the important components in carcinoma-derived exosomes and is most likely to be a key molecule responsible for the down-regulation of molecules such as ZO-1 and VE-cadherin involved in the maintenance of epithelial integrity.

Based on our present results, TSP1 in tumor-derived exosomes plays a pivotal role in the transendothelial migration and possibly metastasis of breast cancer cells. Once TSP1 proteins are released out of the tumor cells as soluble molecules, they are diluted into the surrounding microenvironment and hardly affect the integrity of endothelial cells adjacent to cancer cells. By contrast, tumor-derived exosomes contain a large amount of TSP1 proteins and can efficiently be delivered into adjacent endothelial cells, in which the expression of adhesion molecules are suppressed. Exosomes thus behave as “cancer cell missiles”, which convey a large number of TSP1 molecules, leading to alteration in the expression of junction-related molecules in the target tissues. Interestingly, high TSP1 expression in MCF7 cells promoted the transendothelial migration significantly (Supplementary Figure S6), indicating that breast cancer cells gain the metastatic capability by high TSP1 expression, and TSP1-enriched exosomes further promote the cancer cell transendothelial migration of cancer cells by disrupting the integrity of HUVECs.

LSKL peptide can inhibit TSP-1-mediated activation of TGF-β signaling by interfering with TSP-1 binding to latency-associated peptide (LAP) and blocking the process of converting latent TGF-β in the extracellular matrix to its active form [34]. In the present study, we demonstrated that both HUVEC-derived TSP1 and exosomal TSP1 could inhibit the expression of VE-cadherin and ZO-1 by activating the TGF-β in HUVECs, and that treating HUVECs with LSKL could reverse the inhibition by TSP1 generated from HUVECs themselves, or from exosomal TSP1. Furthermore, our findings also suggested that functional exosomal TSP1 should be localized in the phospholipid bilayer as an integrated membrane protein, but not inside the exosome as a cleaved soluble protein.

For HUVECs treated with exosomes derived from MDA-MB-231/sh-*THBS1* cells, the expression of VE-cadherin and ZO-1 was much higher than that in HUVECs treated with MDA-MB-231-derived exosomes, and even a little bit higher than that in control HUVECs. This implies that certain factors in MDA-MB-231-derived exosomes could promote the expression of these molecules, whereas their effect was overwhelmed by the existence of a high level of TSP1 in MDA-MB-231-derived exosomes. More studies are required to identify and characterize these counteracting factors.

In this study, we employed a zebrafish tumor metastasis model, in which the migration of breast cancer cells with different levels of TSP1 expression was observed. It was shown that breast cancer cells with a high level of TSP1 expression had a tendency to migrate to the tail, demonstrating an in vivo effect of TSP1 on cancer cell migration/metastasis. However, for zebrafish injected with MDA-MB-231/sh-*THBS1* cells, the average number of labeled cancer cells migrated to the tail was similar to that in MDA-MB-231-injected zebrafish, which was not consistent with our in-vitro transendothelial migration results. The discrepancy suggests that TSP1 generated from other cells in zebrafish could compensate the exosomal TSP1 depletion. In our study, exosomal TSP1 derived from breast cancer cells could disrupt the integrity of intercellular junctions, as evidenced by our in vitro (Figure 3 and Figure 5) and in vivo findings (Figure 6). Cancer cells migration to the tail is a combinational result of the changes on intercellular junctions integrity of endothelial cells and the migration activity of cancer cells.

## 4. Materials and Methods

### 4.1. Cell Lines and LSKL Peptide Treatment

HUVEC was purchased from ScienCell Research Laboratories (Carlsbad, CA, USA) with certification and maintained in ECM medium (ScienCell Research Laboratories) with 50 μg/mL streptomycin and 50 IU/mL penicillin in 5% CO_2_ at 37 °C. Human breast cancer cell lines MDA-MB-231 and MCF7 were purchased from and certified by China Center for Type Culture Collection (CCTCC, Wuhan, China) and maintained in RPMI-1640 medium with phenol red (Life Technologies, Carlsbad, CA, USA) supplemented with 10% fetal bovine serum (Tianhang, Hangzhou, China), 50 μg/mL streptomycin and 50 IU/mL penicillin in 5% CO_2_ at 37 °C. LSKL (MCE, NJ, USA), a TSP1 inhibitory peptide, was dissolved in phosphate buffer saline (PBS) and added into HUVEC culture medium to block the function of TSP1.

### 4.2. Preparation of Exosomes-Enriched Precipitate

100 mL of cell culture medium were centrifuged at 3000× *g* to remove cell debris, and culture supernatant was collected for transendothelial migration assay and for the preparation of exosomes-enriched fraction (precipitate) and non-exosome fraction (supernatant) by ultracentrifugation at 100,000× *g*.

### 4.3. Exosomes Preparation

Exosomes were isolated from the cell culture supernatant as follows. After cells and cell debris being removed by centrifugation at 3000× *g* for 30 min, the supernatant was filtrated through a 0.22-μm filter membrane (Millipore, Billerica, MA, USA) and then concentrated using a 10 kDa Microsep concentrator (Millipore). Total Exosome Isolation Reagent (Invitrogen, Carlsbad, CA, USA) was added into the concentrated filtrate and the mixture was allowed to stand at 4 °C overnight. After being centrifuged at 10,000× *g* for 60 min, the supernatant was removed. The exosome-enriched precipitate was collected as exosomes and resuspended in PBS. The final protein concentration of exosome sample was determined by BCA assay.

### 4.4. Characterization of Exosomes

To verify the properties of exosomes by electron microscopy, 10 μL of exosomes were diluted into 1 mL PBS and dropped onto the copper grid coated with carbon membrane. After being treated with 10 μL of 4% glutaraldehyde and then stained with 10 μL of 2% sodium phosphotungstate, the grids with fixed exosomes were air-dried and visualized at 120 kV under a Tecnai G2 F30 transmission electron microscope (FEI, Hillsboro, OR, USA). The size distribution and concentration of exosomes were determined using a ZetaView PMX-120 (Particle Metrix, Meerbusch, Germany), which was equipped with a fast video capture system and a ZetaView software for nanoparticle tracking analysis [35].

### 4.5. Western Blot

Cell lysates were resolved by sodium dodecyl sulfate-polyacrylamide gel electrophoresis (SDS-PAGE) and transferred onto polyvinylidene fluoride (PVDF) membranes (Millipore), which were treated with 5% milk in PBS at room temperature for 1 h. Then the membranes were incubated at 4 °C overnight with anti-TSP1 (Abcam, Cambridge, UK), anti-CD63 (Abcam), anti-TSG101 (Abcam), anti-Calnexin (Abcam), anti-Alix (Abcam), or anti-GAPDH (Cell Signaling Technology, Danvers, MA, USA) antibodies. Then the membranes were treated with fluorescent dye-conjugated secondary antibody and visualized using Odyssey CLx (LI-COR, Lincoln, NE, USA). The strength of the signal for each protein was determined based on the corresponding band intensity of the scanned image.

### 4.6. Immunofluorescence Analysis

Cells were seeded in the glass-bottom cell culture dish (NEST, San Diego, CA, USA). Exosomes were labeled with Dio (Beyotime, Shanghai, China) according to the manufacturer’s instructions. Briefly, exosomes were incubated for 30 min with Dio at a final concentration of 10 μM at 37 °C and re-isolated using the Total Exosome Isolation Reagent Kit (Invitrogen). After being treated with 4% paraformaldehyde, the cells were permeabilized with 0.01% Triton X-100 for 10 min, and then treated with anti-VE-cadherin (Abcam) or anti-ZO-1 monoclonal antibody (Abcam). After being washed, the cells were treated with anti-rabbit fluorescent secondary antibody (Proteintech, Wuhan, China) and fluorescence was observed and photographed using an Olympus FV1000 laser confocal microscope.

### 4.7. Liquid Chromatography-Tandem Mass Spectrometry (LC-MS/MS) Analysis

Exosome sample was subjected to composition analysis using LC-MS/MS at Shanghai Applied Protein Technology Co., Ltd. (Shanghai, China) based on the company’s standard procedure [36]. Exosome samples were digested by trypsin for 20 h, and peptide fragments were extracted. The acidified tryptic peptide fragments were sampled on the Easy-nLC1000 system, which was coupled to the orbitrap Q Exactive mass spectrometry (Thermo/Finnigan). Electrospray ionization (ESI) mass spectrum was used to analyze peptide fragments. After full scan, each intense ion was acquired for further MS/MS scan. The original LC-ESI MS/MS data were subjected to the MASCOT search engine (http://www.matrixscience.com/) for protein identification based on the UniProt database, uniprot_Human_145758_20150518.fasta (145,758 protein sequences, download date of 18 May 2015).

### 4.8. Gene Cloning, Plasmid Construction and Transfection

To obtain breast cancer cell lines with different levels of TSP1 expression, TSP1 knockdown and over expression plasmids were constructed and transfected into breast cancer cells as follows. The short hairpin RNA (shRNA) sequences of *THBS1* were obtained based on the site of Sigma (https://www.sigmaaldrich.com/catalog/genes/THBS1?lang=zh&region=CN). The pLKO.1-puro plasmids containing *THBS1*-specific shRNA were transformed into MDA-MB-231 cells with Lipofectamine 2000 (Invitrogen) to establish TSP1-knockdown cells. After screening with puromycin, *THBS1*-knockdown cell lines were cloned and maintained under the same condition as parent MDA-MB-231 cells. To establish TSP1-overexpressing cell lines, the pcDNA3-*THBS1* plasmids were transformed into MDA-MB-231 or MCF-7 cells with Lipofectamine 2000, and the transfectants were screened with G418 to obtain stable cell lines, MDA-MB-231/*THBS1* and MCF7/*THBS1*, respectively. The primers used for gene cloning and knockdown were as follows: *THBS1*-CDS-F: 5′-CGCAAGCTTATGGGGCTGGCCTGGGGACTA-3′, *THBS1*-CDS-R: 5′-CCGCTCGAGTTAGGGATCTCTACATTCGTA-3′; *THBS1*-KD-F: 5′-CCGGGTAGGTTATGATGAGTTTAATCTCGAGATTAAACTCATCATAACCTACTTTTTG-3′, *THBS1*-KD-R: 5′-AATTCAAAAAGTAGGTTATGATGAGTTTAATCTCGAGATTAAACTCATCATAACCTAC-3′.

### 4.9. Quantitative Real-Time PCR (qRT-PCR)

Total RNA was extracted from cultured cells using TRIZOL (Invitrogen) according to the manufacturer’s instructions. cDNA was synthesized using the First-Strand Synthesis System (Invitrogen) according to the manufacturer’s protocol. The relative levels of mRNA were determined in triplicate using a StepOnePlus Real-Time PCR system (Applied Biosystems). The relative fold changes were calculated using the 2^−ΔΔCT^ method, in which the amplification of β-actin was used for normalization. Primers used for qRT-PCR analyses were listed in Supplementary Table S2.

### 4.10. Transendothelial Migration Assay and Tube Formation

Transendothelial migration assay was performed based on the published protocol [37] with minor modification. First, 200 μL with 4 × 10^4^ HUVECs were seeded into the inserts in a transwell plate (BD Biosciences, Franklin Lakes, NJ, USA) with 8-μm pole-size filters in each well. After 6 h, 200 μL medium was replaced with the equal volume of fresh medium with supernatants (a final protein concentration: 100 μg/mL) or exosomes derived from breast cancer cells (final protein concentration: 0 μg/mL, 10 μg/mL, 100 μg/mL, and 500 μg/mL) and the transwell plates were incubated at 37 °C for 24 h. For control experiments, the equal volume of PBS was added to culture medium instead of exosome samples. Then, 200 μL of fresh medium with 1 × 10^4^ MDA-MB-231 cells were exchanged into transwell inserts with HUVEC cells and 1 mL medium was added into the lower chamber. After being incubated at 37 °C for 48 h, the cells on the top side of the insert transwell membrane were scraped off and those migrated to the bottom side of transwell membrane were examined under an Olympus microscope after being stained with crystal violet. Eight images were recorded randomly at different locations for each membrane. Experiments were repeated three times with three duplications in each batch.

For the tube formation assay, 1 × 10^5^ HUVECs were seeded in 96-well plates pre-coated with Matrigel (BD Biosciences) (40 μL/well, diluted to 1:1 in medium without FBS) and incubated at 37 °C for 24 h in normal culture medium [20,38,39]. The tube formation was observed under an Olympus microscope (CKX41).

### 4.11. In Vivo Metastasis Assay

Wild-type AB strain zebrafish larvae and adults were maintained at 26–28.5 °C under a 14-h light/10-h dark cycle. Fertilized eggs were collected and maintained in E3 medium in an incubator (at ~28.5 °C) for 72 h until the larvae were hatched [40]. Breast cancer cells were stained with Dio (Sigma-Aldrich) according to the manufacturer’s instructions, which were injected into the inferior section of the yolk sac of 48 h post fertilization (hpf) zebrafish and the tumor cell migration was observed and photographed after 48 h under a fluorescent microscope (Olympus SZX16). All animal handling procedures were performed in accordance with protocols approved by the Animal Ethics Committee of Huazhong University of Science and Technology (Ethic Code: S665), and all experiments were conducted according to the relevant guidelines.

### 4.12. Bioinformatics Analysis

Protein candidates obtained from the mass spectral analysis were examined using Gene Ontology biological processes annotation analysis in the DAVID system (https://david.ncifcrf.gov/summary.jsp). The adjusted *p*-value < 0.001 was set as the cut-off criteria to select relevant biological processes for further analysis.

### 4.13. Statistical Analysis

Data were represented as means ± standard deviation. All experiments were representative of at least three independent experiments. Data obtained from different treatment groups were analyzed and graphed using the GraphPad prism (version 6.0) software. Statistical differences between experimental groups were evaluated using Student’s *t*-test.

## 5. Conclusions

**Figure 7:**
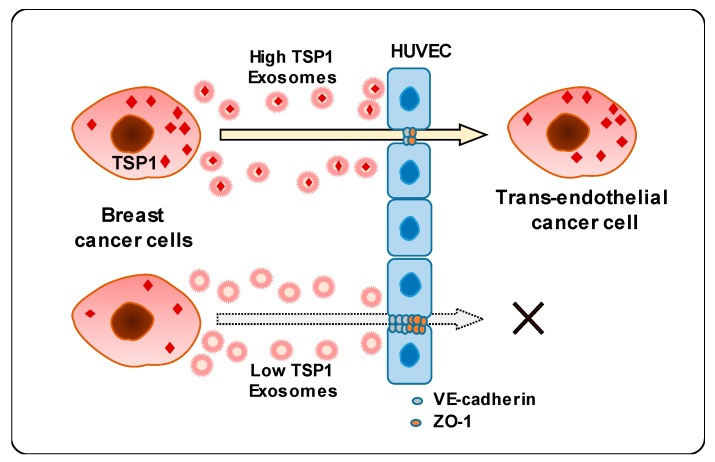
Schematic diagram of transendothelial migration of breast cancer cells with high TSP1 expression via cancer cell-derived exosomes.

This study unveiled a novel mechanism underlying the interaction between breast cancer cell-derived exosomes and endothelial cells. Carcinoma-derived exosomes enhanced the transendothelial migration of breast cancer cells by reducing the expression of intercellular junction proteins. In addition, exosomal proteomic analysis revealed that TSP1 protein was the relevant promising candidate regulating endothelial adhesion and junction. Finally, carcinoma-derived exosomal TSP1 facilitated transendothelial cancer cell migration by decreasing the expression of intercellular junction proteins and disrupting intercellular integrity of endothelial cells both in vitro and in vivo (Figure 7).

## Supplementary Materials

The following file is available online at https://www.mdpi.com/2072-6694/11/12/1946/s1. Table S1: Summary of protein candidates in MDA-MB-231-derived exosome identified by MS, Table S2: Primers used for qRT-PCR, Figure S1: Blank control of transendothelial migration assay, Figure S2: The transendothelial migration of MDA-MB-231 with GW4869 treatment, Figure S3: The cell vitality of HUVECs treated with various amount of exosomes derived from different breast cancer cell lines, Figure S4: TSP1 expression in breast cancer cells and carcinoma-derived exosomes, Figure S5: TSP1 expression in the MDA-MB-231-derived exosomes and cell lysates from different breast cancer cell lines by Western blot, Figure S6: The transendothelial migration of MCF7 and MCF7/*THBS1* cells treated with different TSP1 expression exosomes, Figure S7: TSP1 expression in the exosomes derived from MDA-MB-231 and derivate cells by Western blot.
