# Supplementary material for: Exosomal Thrombospondin-1 Disrupts the Integrity of Endothelial Intercellular Junctions to Facilitate Breast Cancer Cell Metastasis
Source: Cancers (Basel). 2019 Dec 5;11(12):1946. doi: 10.3390/cancers11121946 (PMC6966578; doi:10.3390/cancers11121946)
Supplement: Supplementary file 1 [file cancers-11-01946-s001.zip › Original WB.pdf]

Supplementary Materials for "Exosomal TSP1 disrupts the integrity of endothelial intercellular junctions to facilitate breast cancer cell metastasis"

Original Western blot images for Figures 2 and 4

Figure 2A

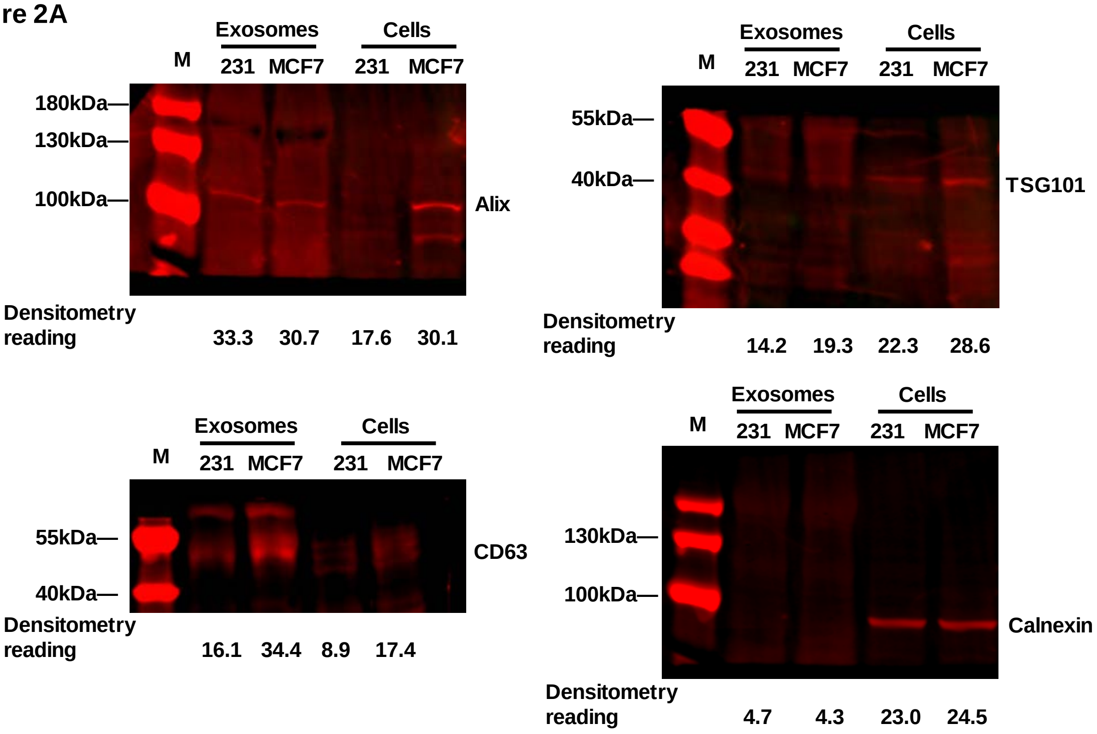

Protein marker (#26616, Thermo Scientific™, Waltham, MA, USA)

Supplementary Materials for "Exosomal TSP1 disrupts the integrity of endothelial intercellular junctions to facilitate breast cancer cell metastasis**"**

Original Western blot images for Figures 2 and 4

Figure 4C

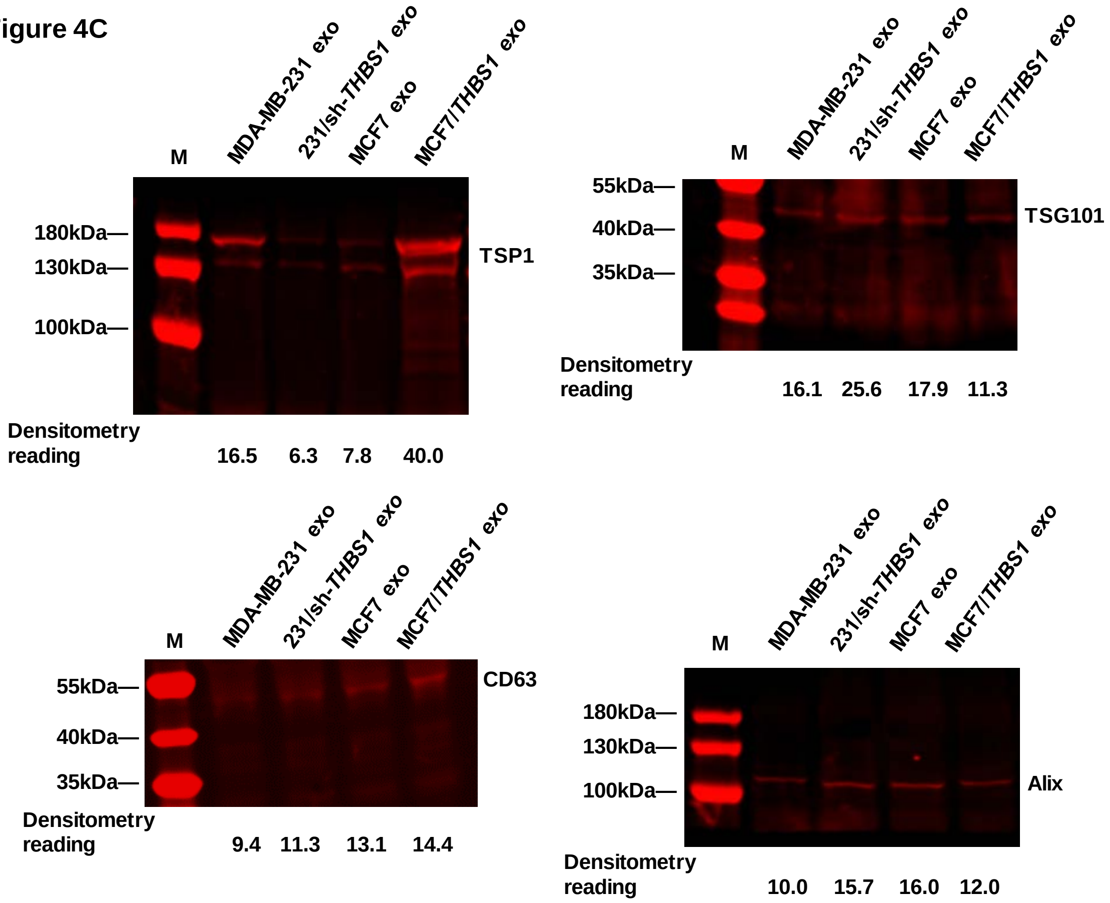

Protein marker (#26616, Thermo Scientific™, Waltham, MA, USA)

Original Western blot images for Supplementary Figures

Figure S3B

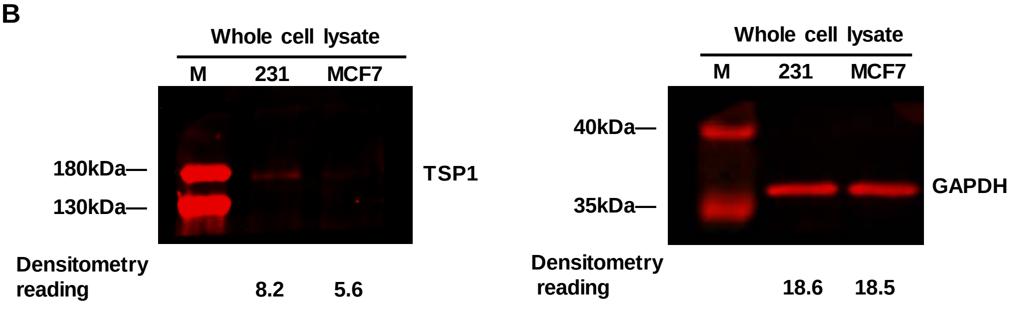

Figure S3C

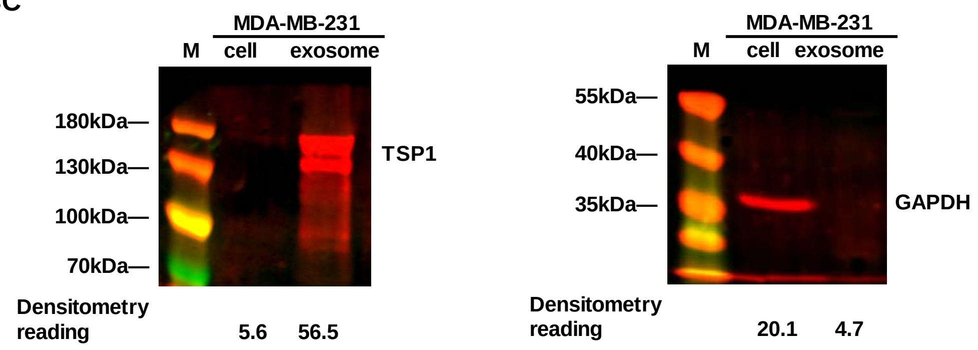

Protein marker (#26616, Thermo Scientific™, Waltham, MA, USA)

Original Western blot images for Supplementary Figures

Figure S4

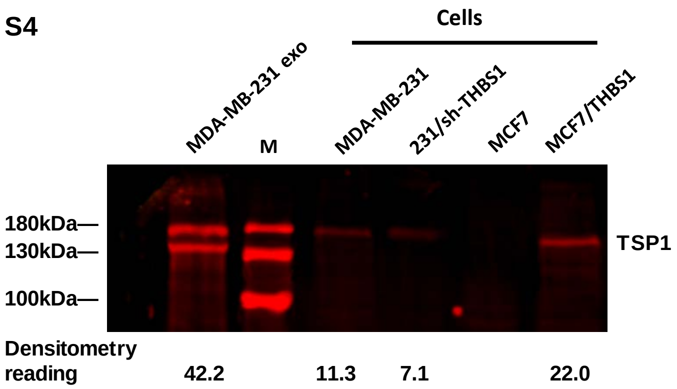

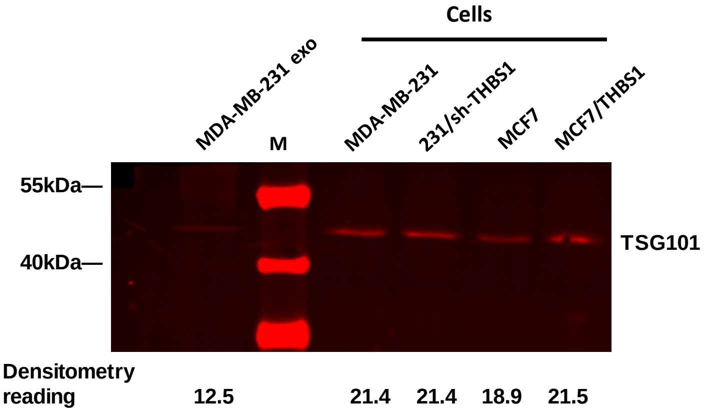

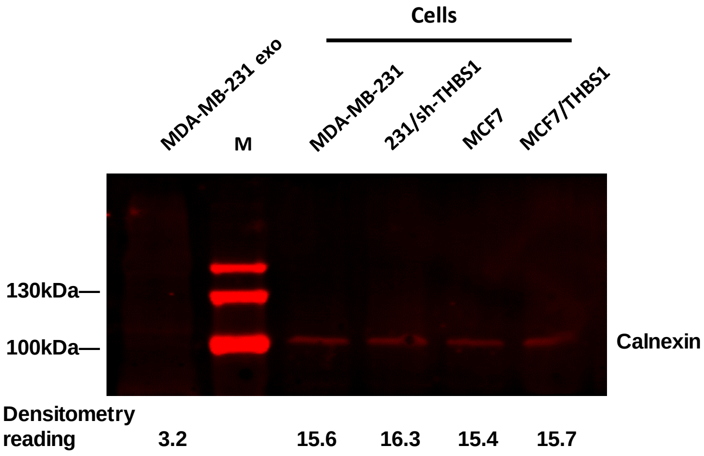

Figure S5

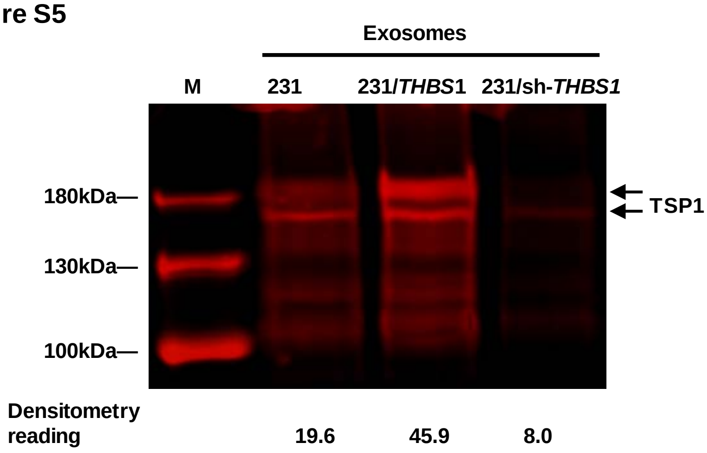

Protein marker (#26616, Thermo Scientific™, Waltham, MA, USA)
