# Supplementary material for: Exosomal Thrombospondin-1 Disrupts the Integrity of Endothelial Intercellular Junctions to Facilitate Breast Cancer Cell Metastasis
Source: Cancers (Basel). 2019 Dec 5;11(12):1946. doi: 10.3390/cancers11121946 (PMC6966578; doi:10.3390/cancers11121946)
Supplement: Supplementary file 1 [file cancers-11-01946-s001.zip › Supplementary Materials.pdf]

cancers

MDPI

Supplementary Materials

Exosomal Thrombospondin-1 Disrupts the Integrity of Endothelial Intercellular Junctions to Facilitate Breast Cancer Cell Metastasis

Junyu Cen, Lingyun Feng, Huichuan Ke, Lifeng Bao, Lin Z. Li, Yoshimasa Tanaka, Jun Weng and Li Su

Table S1. Summary of protein candidates in MDA-MB-231-derived exosome identified by MS.

UniPepCount	ProteinGroupCount	Percent	ProteinCount	Percent
1	263	83.49%	1106	84.11%
2	29	9.21%	94	7.15%
3	11	3.49%	70	5.32%
4	3	0.95%	5	0.38%
5	4	1.27%	22	1.67%
6	2	0.63%	10	0.76%
7	1	0.32%	3	0.23%
8	1	0.32%	4	0.30%
18*	1	0.32%	1	0.08%
**Total**	**315**		**1315**	

* The protein candidate matched with 18 unique peptides identified in MS is TSP1.

Table 2. Primers used for qRT-PCR.

Name	Sequences
VE-cadherin-F	5′- ATGTAGGCAAGATCAAGTCAAG-3′
VE-cadherin-R	5′-CCTCTCAATGGCGAACAC-3′
ZO-1-F	5′-CAACATACAGTGACGCTTCACA-3′
ZO-1-R	5′-CACTATTGACGTTTCCCCACTC-3′
Occludin-F	5′-GACTTCAGGCAGCCTCGTTAC-3′
Occludin-R	5′-GCCAGTTGTGTAGTCTGTCTCA-3′
MCAM-F	5′-AATATGGTGTTGAATCTGTCTTG-3′
MCAM-R	5′-GGCTAATGCCTCAGATCGATG-3′
MIC2-F	5′-GCCAAATCCAAACCCCAACC-3′
MIC2-R	5′-CACCTCCCCTTGTTCTGCAT-3′
GJA1-F	5′-GAGGTGGCCTTCTTGCTGAT-3′
GJA1-R	5′-ACCACTGGTCGCATGGTAAG-3′
N-Cad-F	5′-TCAGGCGTCTGTAGAGGCTT-3′
N-Cad-R	5′-ATGCACATCCTTCGATAAGACTG-3′
PECAM-F	5′- GTCAAGCCTCAGCACCAGAT-3′
PECAM-R	5′-CACCTGGTACTCTGCAGTGG -3′
ZO-2-F	5′-GGGAAGGTCGCTGCTATTGT-3′
ZO-2-R	5′-CTCTCGCTGTAGCCACTCC-3′
β-actin-F	5′-CGGAACCGCTCATTGCC-3′
β-actin-F	5′-ACCCACACTGTGCCCATCTA-3′

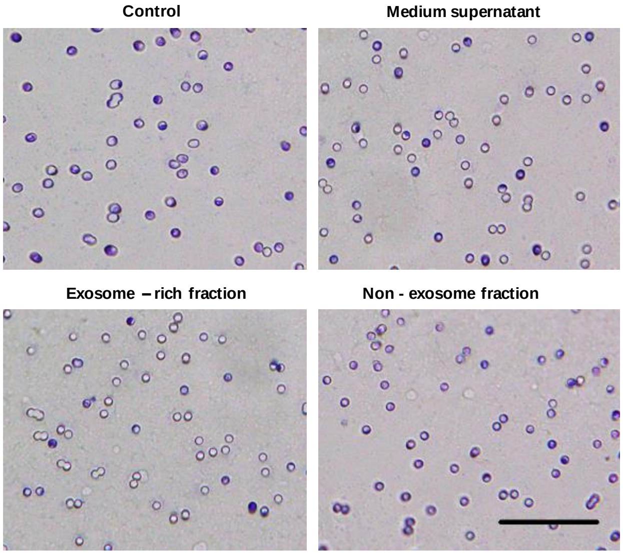

Figure S1. Blank control for trans-endothelial migration assay. The experimental process is the same as Figure 1B, except that the medium with MDA-MB-231 cells was repalced with no-carcinoma-cell medium added in the upper chamber of transwell. Circles in the images show the poles in the transwell membranes. Scale bar, 100 μm.

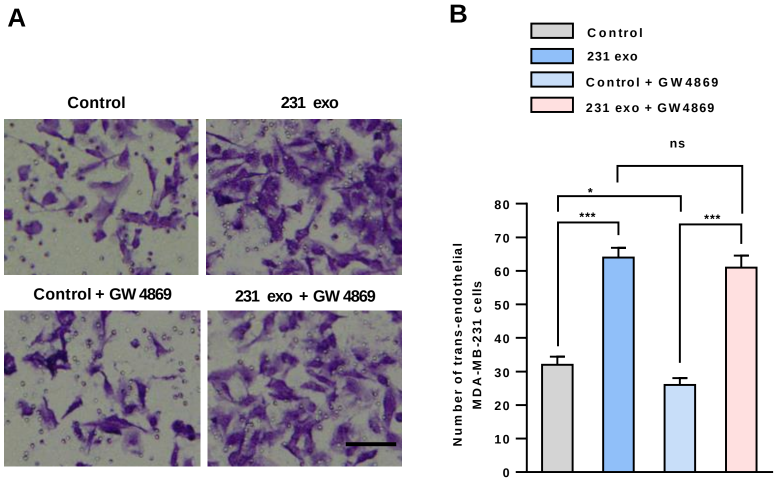

Figure S2. The trans-endothelial migration of MDA-MB-231 with GW4869 treatment. (**A**) Trans-endothelial migration of MDA-MB-231 cells after treating HUVECs with MDA-MB-231-derived exosomes. 10μM GW4869 was added with MDA-MB-231 cells. Scale bar, 100 μm. (**B**) Quantitative analyses of the migrated MDA-MB-231 cells in the trans-endothelial assay with GW4869 treatment. Data are shown as mean ± SD and representative of three independent experiments. ** $p < 0.01$, *** $p < 0.001$ by unpaired Student's t-test.

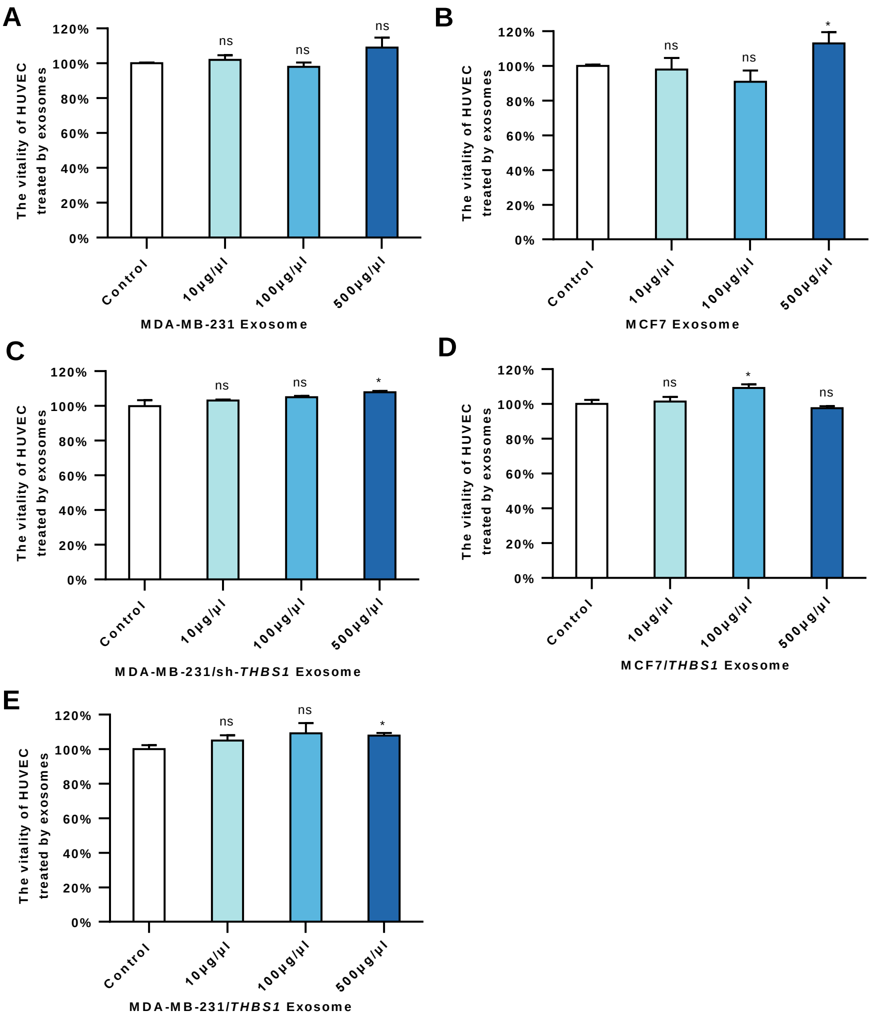

Figure S3. The cell vitality of HUVECs treated with various amount of exosomes derived from different breast cancer cell lines. HUVECs were treated with breast cancer cell-derived exosomes for 48h and the cell vitality of HUVECs was detected by CCK8 kit. * $p < 0.05$; ns, no significance by unpaired Student's *t*-test.

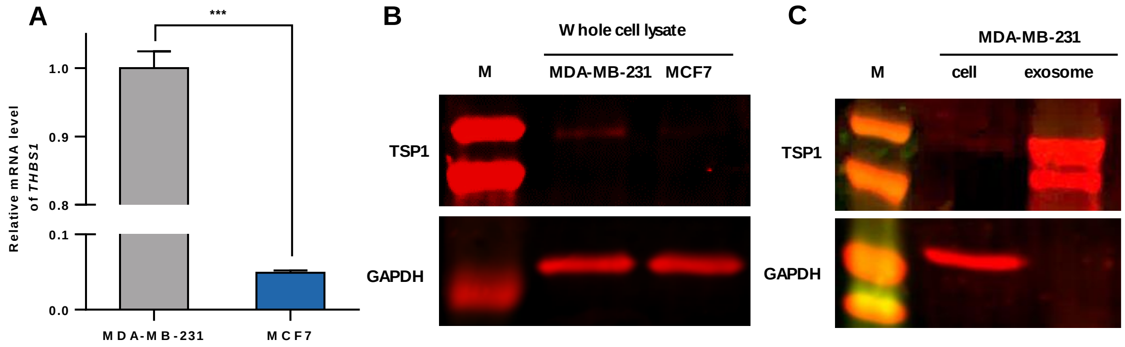

Figure S4. TSP1 expression in breast cancer cells and carcinoma-derived exosomes. (A) mRNA expression of MDA-MB-231 and MCF7 cells by qRT-PCR analysis. (B) TSP1 expression in MDA-MB-231 and MCF7 cells by Western blot. (C) TSP1 expression in MDA-MB-231 cells and exosomes by Western blot. Data are shown as mean ± SD and representative of three independent experiments. *** $p < 0.001$ by unpaired Student's *t*-test.

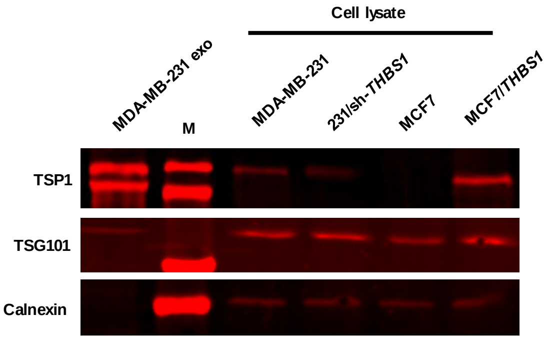

Figure S5. TSP1 expression in the MDA-MB-231-derived exosomes and cell lysates from different breast cancer cell lines by Western blot.

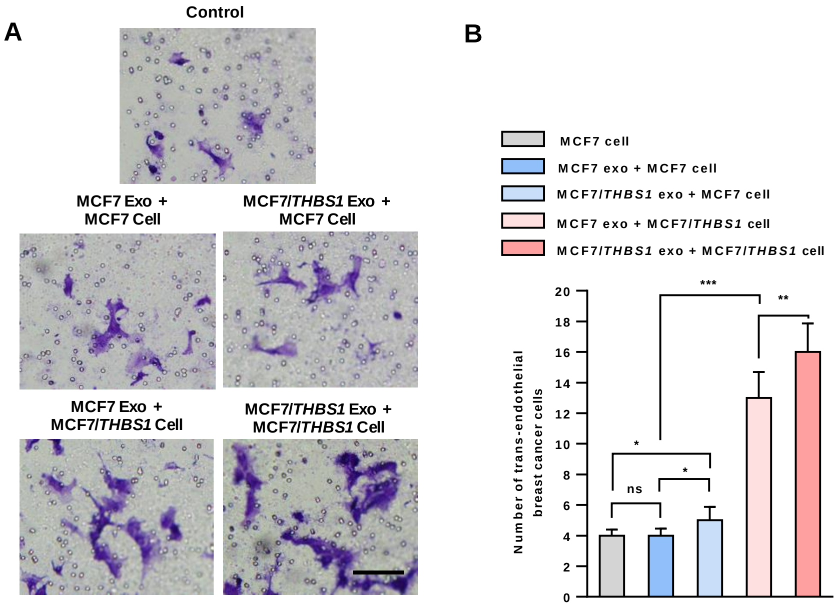

Figure S6. The trans-endothelial migration of MCF7 and MCF7/*THBS1* cells treated with different TSP1 expression exosomes. (**A**) Trans-endothelial migration of MCF7 and MCF7/*THBS1* cells after treating HUVECs with the exosomes with different TSP1 expressing. Scale bar, 100 μm. (**B**) Quantitative analyses of the migrated MCF7 and MCF7/*THBS1* cells in the trans-endothelial assay. Data are shown as mean ± SD and representative of three independent experiments. ** $p < 0.01$, *** $p < 0.001$ by unpaired Student's *t*-test.

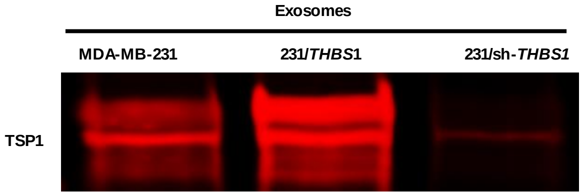

Figure S7. TSP1 expression in the exosomes derived from MDA-MB-231 and derivate cells by Western blot.
